# Oral Intake of Collagen Peptide Attenuates Ultraviolet B Irradiation-Induced Skin Dehydration In Vivo by Regulating Hyaluronic Acid Synthesis

**DOI:** 10.3390/ijms19113551

**Published:** 2018-11-11

**Authors:** Min Cheol Kang, Silvia Yumnam, Sun Yeou Kim

**Affiliations:** College of Pharmacy, Gachon University, 191, Hambakmoero, Yeonsu-gu, Incheon 21936, Korea; mincjf07@gmail.com (M.C.K.); silviayumnam@gmail.com (S.Y.)

**Keywords:** Photoaging, ultraviolet B, Collagen peptide, Skin hydration, Hyaluronic acid, SKH-1

## Abstract

Collagen peptide (CP) has beneficial effects on functions of the skin, such as skin barrier function and skin elasticity, in vivo. However, there are few studies investigating the mechanism underlying the potential effects of CP in skin epidermal moisturization after ultraviolet B (UVB) irradiation. In this study, we examined whether orally-administered CP affects the loss of skin hydration induced by UVB irradiation in hairless mice. SKH-1 hairless mice were orally administered CP at two doses (500 and 1000 mg/kg) for nine weeks, and the dorsal skin was exposed to UVB. The potential effects of CP were evaluated by measuring the transepidermal water loss (TEWL), skin hydration, wrinkle formation, and hyaluronic acid expression in the dorsal mice skin. We found that oral administration of CP increased skin hydration and decreased wrinkle formation compared to the UVB-irradiated group. Treatment of CP increased the mRNA and protein expression of hyaluronic acid synthases (HAS-1 and -2) concomitant with an increased hyaluronic acid production in skin tissue. The expression of hyaluronidase (HYAL-1 and 2) mRNA was downregulated in the CP-treated group. In addition, the protein expression of skin-hydrating factors, filaggrin and involucrin, was upregulated via oral administration of CP. In summary, these results show that oral administration of CP increases hyaluronic acid levels, which decreases during UVB photoaging. Therefore, we suggest that CP can be used as a nutricosmetic ingredient with potential effects on UVB-induced skin dehydration and moisture loss in addition to wrinkle formation.

## 1. Introduction

Repetitive exposure to ultraviolet radiations (UVRs) leads to various skin disorders including inflammation, aging, and cancer [1]. UVRs are divided into three types according to their wavelength: UVA (315–400 nm); UVB (280–315 nm); and UVC (100–280 nm). UVB irradiation is known as a mutagenic and cancerous factor that can penetrate and change the structure of skin cells. Long-term overexposure to UVB usually induces photoaging [2] and damages the skin epidermis and dermis. Continuous UVB exposure induces wrinkle formation, epidermal skin thickness, irregular hyperpigmentation, and laxity [3]. Overexposure to UVB also increases transepidermal water loss (TEWL) and decreases skin hydration, which impairs skin barrier function and aggravates skin wrinkling [4].

Recently, the protection and treatment of skin conditions using dietary supplements have received increasing attention. There have been many efforts to find natural resources that have protective effects against UVB-induced skin deterioration. The identification of a suitable candidate for dietary supplements can help protect against UVB-induced skin deterioration and maintain healthy skin in healthy people. Oral supplementation with food ingredients including vitamins and antioxidants, fatty acids, and hydrolyzed proteins helps improve skin functions damaged by overexposure of UVB [5].

This trend is observed across all nutricosmetics key industries. An example is skin-derived collagen peptides (CPs) that ameliorate UVB-induced skin aging. Collagen is a group of naturally occurring proteins and is one of the major constituents of the connective tissue of animals, birds, and fishes. Collagen in the body is classified into type I, II, III, and IV [6]. Porcine skin, bovine skin, and tendon are the major source of collagen but recently, marine collagen is used as an alternative source of collagen [7]. Fishes, mollusks, and sponges are the major source of marine collagen. Collagen extracted from scales of the *Oreochromis* species is mainly Type I collagen [8]. When collagen is heated, it becomes water-soluble gelatin, a denatured form of collagen [9]. CPs are prepared via enzymatic hydrolysis of gelatin at an industrial scale [10]. CPs have been used in pharmaceuticals and food supplements for improving skin dysfunction and loss of cartilage tissues. CP is usually absorbed in the digestive tract and is accumulated in the skin tissue for 96 h [11,12]. It also shows several biological functions such as chemotaxis, cell proliferation [13], and antioxidation [14]. CP from the skin of *Oreochromis niloticus* (Tilapia) has been reported to enhance wound healing [15]. CPs have been reported to be beneficial for skin dysfunction in UVB-induced skin aging models [16]. In addition, recent studies have shown that collagen hydrolysate intake increases skin collagen expression and inhibits matrix metalloproteinase 2 activity, a significant contributing factor responsible for wrinkle formation, which decreases during skin photoaging [17]. Thus, the results of previous studies imply a possibility that CPs may provide multifunctional activity for photodamaged skin.

Although there is evidence for the beneficial effects of CPs on UVB irradiation-induced skin dysfunction in humans or animals, the effect of CP on skin hydration and the underlying mechanisms are poorly understood. To determine the skin-hydration and anti-wrinkle effects of oral treatment with CP, we investigated its activities and mechanisms in UVB-irradiated SKH-1 hairless mice skin models.

## 2. Results

### 2.1. Changes in Transepidermal Water Loss, Skin Hydration, and Epidermal Thickness in the Skin of Hairless Mice Treated with Collagen Peptide

**Figure 1:**
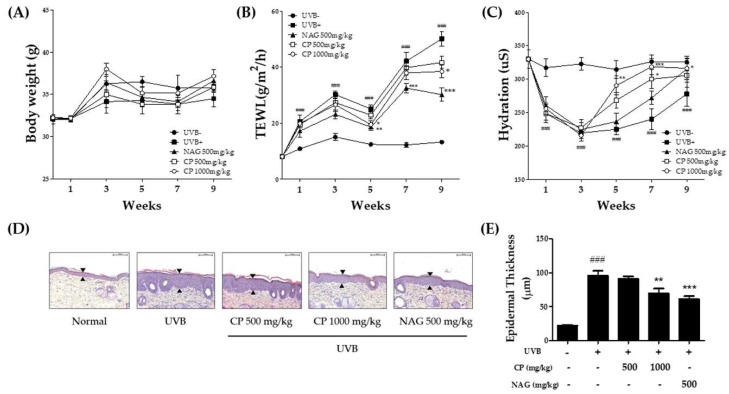
Effect of oral administration of collagen peptide on TEWL, hydration, and epidermal thickness in the dorsal skin of UVB-irradiated hairless mice. (**A**) Body weight changes, (**B**) TEWL, and (**C**) stratum corneum water content after repeated irradiation with UVB were measured using the Derma Combo System; (**D**) hematoxylin and eosin staining. Scale bar = 100 μm. Arrows indicate the thickness of epidermis; (**E**) histogram of hematoxylin and eosin staining. The values are shown as mean ± SEM (*n* = 8). ### *p* < 0.001 (vs. control mice). * *p* < 0.05, ** *p* < 0.01, and *** *p* < 0.001 (vs. UVB-irradiated mice).

Basal TEWL and stratum corneum hydration were measured to investigate the effects of CP on epidermal barrier function. During the experiment period, the body weights of mice were similar between the groups (Figure 1A). TEWL and stratum corneum water content are shown in Figure 1B,C. TEWL and epidermal hydration were significantly different in UVB-treated groups. TEWL was low in CP 1000 mg/kg-treated group and *N*-acetyl glucosamine (NAG) group after five weeks. Five weeks after treatment, a significant difference in the stratum corneum water content was observed in both CP 500 mg/kg and 1000 mg/kg treated groups. NAG increased skin hydration effects compared with the collagen groups. Based on histological examination results, it was found that oral treatment of NAG increased epidermal thickness in UVB-irradiated mice compared to control mice (Figure 1D). UVB-irradiated mice treated with CP 1000 mg/kg showed significant recovery from these pathological changes compared with UVB-irradiated mice (Figure 1E).

### 2.2. Hyaluronic Acid Production and HAS and HYAL mRNA and Protein Expression in the Skin Tissue of UV-Irradiated Hairless Mice Treated with Collagen Peptide

**Figure 2:**
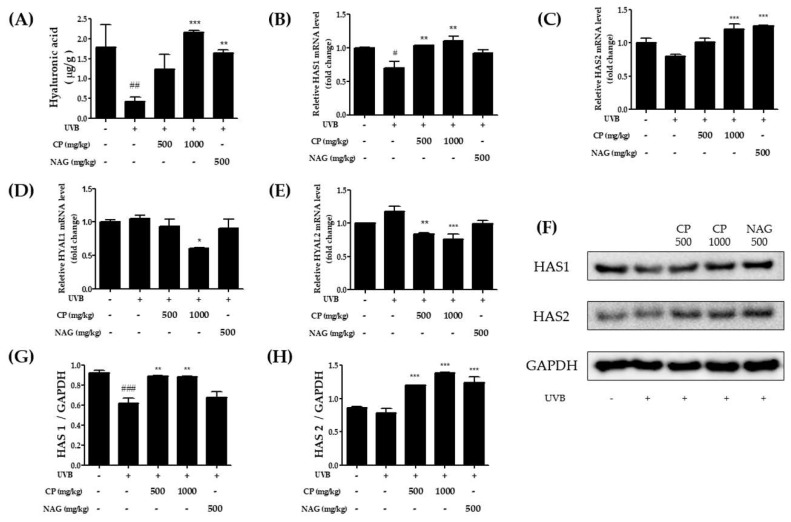
Effects of oral administration of collagen peptide on hyaluronic acid content, HAS isoenzymes, and hyaluronidase mRNA and protein expression in UVB-irradiated hairless mice. (**A**) Hyaluronic acid content in the dermis 9 weeks after UVB irradiation. mRNA levels of (**B**) HAS1, (**C**) HAS2, (**D**) HYAL1, and (**E**) HYAL2 were estimated by real-time PCR; (**F**) Western blotting and (**G**,**H**) relative densities of HAS1 and HAS2. The values are shown as mean ± SEM (*n* = 8). # *p* < 0.05, ## *p* < 0.01, and ### *p* < 0.001 (vs. control mice). * *p* < 0.05, ** *p* < 0.01, and *** *p* < 0.001 (vs. UVB-irradiated mice).

The dermal HA content after nine weeks of UVB irradiation is shown in Figure 2A. The total dermal HA per mg dry weight significantly decreased compared with the non-irradiated control group. The HA content was significantly higher in the CP-treated group than in the positive NAG treated group. Figure 2B–E shows the effects of CP on the mRNA levels of HAS and hyaluronidase (HYAL) in the skin of hairless mice irradiated with UVB. A high dose of CP oral treatment significantly increased HAS1 and HAS2 mRNA expression compared with the UVB group. Similarly, the HAS protein levels increased after UVB irradiation (Figure 2G,H). In contrast, the CP treatment significantly decreased both HYAL1 and HYAL 2 levels, although no changes were observed between the non-UV irradiated group and UV irradiated group. These results suggest that repetitive UVB irradiation for 9 weeks decreased HA production from the upper dermis. However, CP treatment recovered the loss of HA via regulation of HAS1, HAS2, and HYAL 2.

### 2.3. Expression of Skin Barrier Function-Related Proteins in the Dorsal Skin of Hairless Mice Treated with Collagen Peptide

**Figure 3:**
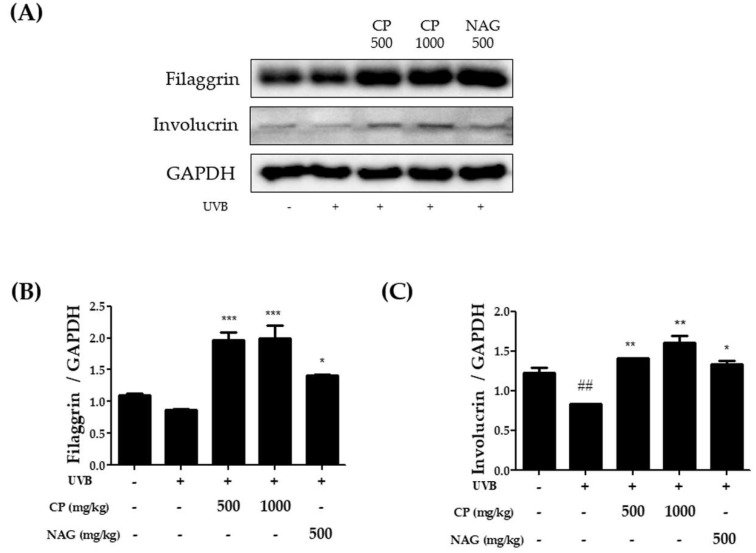
Effect of oral administration of collagen peptide on the expression of filaggrin and involucrin in the dorsal skin of UVB-irradiated mice. (**A**) Western blotting and (**B**,**C**) relative densities of filaggrin and involucrin. The values are shown as mean ± SEM (*n* = 8). ## *p* < 0.01 (vs. control mice). * *p* < 0.05, ** *p* < 0.01, and *** *p* < 0.001 (vs. UVB-irradiated mice).

To determine the role of CP in skin barrier function, the protein expression of filaggrin and involucrin was evaluated using western blot analysis. Figure 3 shows that skin barrier function was reduced in the UVB irradiated group when compared to the non-irradiated group. However, this reduction was significantly increased as that the protein expression of filaggrin and involucrin were upregulated in CP-treated groups (Figure 3). Also, the expression of both filaggrin and involucrin were higher in the CP treated group than that of the NAG treated group. These results suggest that dietary CP may protect the skin barrier in UVB-induced skin dysfunction models.

### 2.4. Changes in Wrinkle Formation in the Dorsal Skin of Hairless Mice Treated with Collagen Peptide

**Figure 4:**
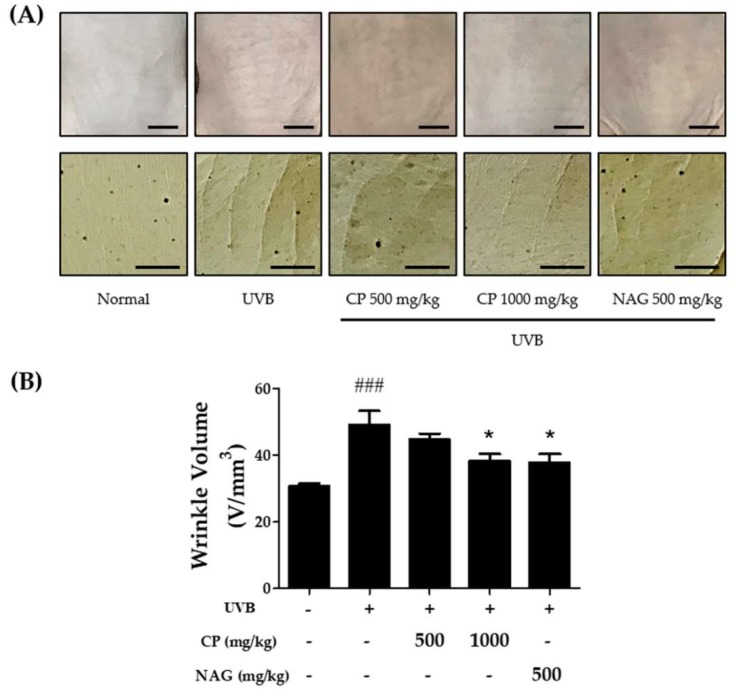
Effect of oral administration of collagen peptide on visible skin condition and wrinkle formation in UVB-induced hairless mice. (**A**) Photographs of a skin surface and replica impression of the mouse dorsal skin. Scale bar = 5 mm; (**B**) histogram of dorsal wrinkle using a video camera and calculated image analysis. The values are shown as mean ± SEM (*n* = 8). ### *p* < 0.001 (vs. control mice). * *p* < 0.05 (vs. UVB-irradiated mice).

Figure 4A shows visible wrinkle formation on the skin of mice treated with CP or UVB. UVB irradiation induced deep, coarse furrows and wrinkles in the dorsal skin when compared with the control group. However, a reduction in roughness of wrinkles was observed in mice treated with CP compared with that in mice exposed to UVB alone. The effect of CP treatment on wrinkle appearance was also examined using image analysis for quantifying the skin surface (Figure. 4B). Based on image analysis of the dorsal skin, we can suggest that CP 1000 mg/kg may attenuate UVB-induced wrinkle formation.

## 3. Discussion

Skin aging is divided into chronological aging and UVR irradiation-induced photoaging [18]. Chronic exposure to sun causes photoaging, which differs between sun-protected skin and sun-exposed skin. Photoaging is characterized by decreased skin barrier function, wrinkles, dryness, roughness, and pigmented spots [19]. In this study, we aimed to determine the antiaging and skin hydration effects of dietary CP on UVB irradiation-induced photoaging in mice and to elucidate the underlying mechanisms. We found that the administration of CP significantly reduced the abnormalities of the epidermal barrier and skin hydration after UVB irradiation. Our study showed that dietary CP increased the expression of natural moisturizing factor (NMF)-related genes filaggrin (FLG) and involucrin (IVC), and the mRNA and protein expression of HAS1 and HAS2 under UVB irradiation conditions. In addition, the expression level of HYAL 1 and HYAL 2 was decreased in UVB-treated dorsal skin tissues via oral treatment of CP. Furthermore, we confirmed the anti-wrinkle activity of CP and demonstrated its protective effects against UVB skin damage via the downregulation of HA production.

Skin hydration is critical for maintaining healthy skin, and moisturizers are an essential component of primary skin care. Stratum corneum plays the role of a skin barrier and prevents water loss. The role of NMF within corneocytes and the importance of stratum corneum intercellular lipid organization to form a barrier to TEWL have been reported [20]. In this study, UVB irradiation significantly increased TEWL and decreased skin hydration. UVB-irradiated barrier dysfunction was also related to a hyper-proliferation activity in epidermal keratinocytes. It has been presumed that the administration of CP might affect TEWL and skin hydration of UV-damaged mouse skin. CP effectively inhibited TEWL and recovered the low level of skin hydration caused by UVB irradiation-induced dorsal skin dysfunction.

High levels of skin hydration are required for proper skin barrier function. In the extracellular matrix (ECM) of the skin, HA plays an essential role in hydration balance owing to its water-holding properties [21]. In the dermis, HA contributes to the hydration and plasticity of the skin. HA is a high-molecular-weight polysaccharide composed of repeating polymeric disaccharides of d-glucuronic acid and *N*-acetyl-d-glucosamine, which are major components of the extracellular matrix [22]. HA plays a key role in the metabolism of the dermis and in providing tissue hydration and structural integrity to the dermis.

HA is also naturally present in the epidermis. It binds to the extracellular space via CD44 and may play a role in epidermal barrier function and hydration [23]. Three HAS (HAS-1, -2, -3) are involved in the synthesis of HA at the inner plasma membrane [24]. Degradation of HA is achieved through the HYAL family, HYAL-1 and -2, which are acid-active enzymes located in the plasma membrane [25]. It has been reported that UVB irradiation induces loss of HA from the dermis and leads to the quiescent phenotype of dermal fibroblasts, such as inhibition of migration and proliferation [26]. Our results showed that the protein and mRNA expression of HAS1 and HAS2 decreased in UVB-induced mice skin. The decreased level of HA is probably due to reduced HA synthesis and not decreased degradation, because no decrease in HYAL mRNA expression was observed compared with the non-irradiated control group. Administration of CP effectively increased HA content after UVB irradiation in the dermis. We also investigated the effects of CP on the expression of HAS or HYAL. CP oral treatment significantly increased both HAS1 and HAS2 protein levels compared with the control group. The mRNA expression of HAS was also increased in the CP-treated group. The mRNA and protein expression of HAS2 also increased dose-dependently. Although there were no significant changes in HYAL between the non-irradiated group and irradiated group, CP treatment slightly reduced HYAL2 mRNA expression in UVB-induced mice skin, while mRNA level of HYAL1 did not show any significant change. Therefore, we presumed that dermal HA production and HAS expression recovered by CP may be involved in the increase of HAS isoform expression in UVB-irradiated mice skin. In addition, the level of HA and its expression may be related to the increased stratum corneum water content in UVB-irradiated mice skin after 5 weeks of CP administration.

The critical factor to maintain skin hydration is to improve the skin barrier function. Skin barrier dysfunction can be influenced by the dysregulation of factors such as involucrin and filaggrin [27]. Filaggrin is then degraded into free amino acids, which are a major component of NMFs [28]. Involucrin consists of repeating peptide units, which is a component of the cornified envelope in the epidermis [29]. UVB irradiation can cause dysfunction of the skin barrier and induce water loss via changes to the protein expression of involucrin and filaggrin [30,31]. UVB irradiation can cause dysfunction of the skin barrier and induce water loss via changes to the protein expression of involucrin and filaggrin. In our study, we observed that UVB significantly downregulated the expression of filaggrin and involucrin, which correlated with TEWL, while CP upregulated the expression of filaggrin and involucrin after 5 weeks. Furthermore, the epidermal thickness of mice treated with CP reduced by almost half compared to that of UVB-irradiated mice skin. Therefore, it seems that CP may restore the skin barrier and help to maintain skin hydration function in UVB damaged skin. Skin elasticity is affected by UVB exposure. Chronic UVB exposure leads to alterations in dermal structure and elasticity. Elasticity is also closely related to the dermal components, especially ECM. ECM is composed of dense collagen (Types I and III) and elastin, and embedded proteoglycans, such as HA, that maintain the structural and functional integrity of interstitial ECM [32]. In the skin, photodamaged- and chronologically aging lead to a decrease in the synthesis and changes in the arrangement of elastin and collagen, in addition to the loss of glycosaminoglycans, which are responsible for the integrity of skin tissues. Namely, skin tissue quality is affected by age-related changes in skin, collagen, and its cross-linking profiles. Therefore, loss of collagen synthesis is a characteristic feature of photodamaged and chronologically aged skin [33].

Moreover, the absence of HA leads to fine skin lines and wrinkles. HA may contribute to the disorganization of elastin fibers and collagen [34]. Hydration of the skin leads to the slow down and improvement of wrinkle formation. UVB irradiation significantly induced wrinkle formation on the dorsal skin. However, oral administration of CP ameliorated wrinkle formation induced by UVB irradiation. It has been reported that skin rejuvenation and anti-wrinkle effects of HA are caused by stimulated collagen synthesis via induction of fibroblasts in the dermis [35]. The increase of collagen via CP intake makes the skin smoother, reduces wrinkles, and improves skin elasticity. Notably, regulation of HA production via oral administration of CP at doses of 500 and 1000 mg/kg body weight/day increased skin hydration and reduced wrinkle formation in UVB-damaged skin condition.

In summary, our results suggest that oral administration of CP might attenuate UVB-induced wrinkle formation and repair skin barrier function in hairless mice. Thus, CP will be a potential bioactive ingredient in nutricosmetics with beneficial effects on skin photoaging via regulation of HA.

## 4. Materials and Methods

### 4.1. Animals

Specific pathogen-free 6-week-old male SKH-1 hairless mice were purchased from Orient Bio (Gyeonggi-do, Korea). All mice were acclimated for one week before the experiment in a temperature- and humidity-controlled room (23 ± 1 °C and 60 ± 5% humidity) under 12/12 light/dark cycles. All experimental procedures for animal experiments were reviewed and approved by the animal care committee of the Center of Animal Care and Use (CACU, LCDI-2018-0010, 06. 02. 2018) at the Lee Gil Ya Cancer and Diabetes Institute, Gachon University, Korea.

### 4.2. UVB Radiation

SKH-1 hairless mice were subjected to UV irradiation using a UV irradiation system (BIO-LINK^®^ BLX-312, Vilber Lourmat, Collegien, France) that emitted wavelengths with a peak emission at 312 nm. The intensity of UV irradiation was determined as 150 mJ/cm^2^ using a radiometer (VLX-3W, UVItec). The UV dose used in this study was a sub-erythema dose (just below 1 minimalerythemal dose, MED), and the dorsal skin of mice was exposed to UVB radiation two times a week for eight weeks.

### 4.3. Treatment

The CP used in this study is hydrolysate extracted from tilapia scale (*Oreochromis mossambicus*). The CP was manufactured by Nitta Gelatin India Limited Co. (Cochin, India, Product: KER-30DR), and it was obtained from Ju Yeong NS (Seoul, Korea). After acclimatization for one week, the mice were randomly divided into five groups of eight mice each according to body weight, TEWL, and hydration of stratum corneum (Table 1) as follows: (1) normal group (control); (2) UVB-irradiated group (UVB control); (3) UVB-irradiated and 500 mg/kg/day CP-treated group; (4) UVB-irradiated and 1000 mg/kg/day CP-treated group; and (5) UVB-irradiated and 1000 mg/kg/day N-acetyl glucosamine (Sigma-Aldrich, St. Louis, MO, USA) -treated group as a positive control [36]. The non-irradiated control group was given 10 mL/kg/day while the UVB irradiated group was given 10 mL/kg/day of the sample as indicated in Table 1 using a stomach sonde needle (φ1.2 × 50 mm). The mice in the treated groups received oral CP daily for 9 weeks with concurrent exposure to UVB radiation three times per week.

### 4.4. Evaluation of Skin Barrier Function

TEWL in mouse dorsal skin was measured under specific conditions of 21–22 °C and 50–55% humidity using a Dermalab Combo system (C40000.03-189, Cortex Technology, Hadsund, Denmark) once a week. The measurements were recorded when TEWL readings stabilized at approximately 30 s after the probe was placed on the skin. The data were analyzed with a microprocessor and expressed in g/m²/h. Hydration of the stratum corneum was also measured using a Dermalab Combo system. The instrument is based on the measurement of high-frequency electrical conductance. Three independent measurements of the same skin area were averaged for each value.

### 4.5. Wrinkle Measurement

Skin condition was assessed by photographing the mouse dorsal skin at the end of the experiment to measure the formation of wrinkles. Skin replicas were prepared using a SILFLO kit (CuDerm Corporation, Dallas, TX, USA). The skin surface was analyzed using Visioscan^®^ VC 98/software SELS 2000 (Courage & Khazaka, Cologne, Germany).

### 4.6. Western Blot Analysis

The animals were sacrificed after the final UVB exposure, and mouse dorsal skins were collected and stored at −80 °C. The harvested tissues were homogenized in Pro-prep solution (iNtRON Biotechnology, Seoul, Korea) and lysates were centrifuged at 12,000× *g* for 30 min. Total protein (30 μg) was separated on 12% sodium dodecyl sulfate (SDS)-polyacrylamide gel electrophoresis (PAGE) gels and transferred to polyvinylidene fluoride (PVDF) membranes (Millipore, MA, USA). After the transfer, the membranes were blocked with a 5% fat-free milk-TBST buffer for 1 h and washed with TBS containing a 0.05% Tween-20 (TBST) buffer. The membrane was incubated overnight at 4 °C with 1:1000 dilutions of primary antibodies for hyaluronic acid synthase (HAS) 1, HAS2, filaggrin, and involucrin. The blots were washed three times with TBST and incubated with secondary antibodies for 1 h at room temperature. The transferred proteins were visualized with a Pierce ECL Western blotting substrate (Thermo Scientific, Rockford, IL, USA).

### 4.7. RNA Isolation and Quantitative Real-Time Polymerase Chain Reaction (PCR) Analysis

Total RNA was extracted from the dorsal skin tissues and isolated using the Trizol^®^ reagent (Invitrogen, Carlsbad, CA, USA) according to the manufacturer’s instructions. cDNA synthesis was performed according to the instructions of Takara PrimeScript™ RT reagent kit (Takara, Tokyo, Japan). For real-time PCR, TB Green^®^ Premix Ex Taq™ II (Takara, Tokyo, Japan) was used, and all reactions were repeated three times with the following conditions: initial denaturation at 95 °C for 30 s, followed by 40 cycles at 95 °C for 5 s and 60 °C for 30 s. The primers used for real time-PCR are listed in Table 2.

### 4.8. Histological Skin Analysis

The collected tissues were fixed in 10% buffered-neutral formalin for 24 h and embedded in paraffin. Five-micrometer-thick sections were stained with hematoxylin for 10 min, PBS washed, and then stained with eosin for 2 min. The slides were visualized under a light microscope (Olympus, Tokyo, Japan).

### 4.9. Extraction and Quantification of Hyaluronic Acid

Dorsal skin tissues were separated into dermis and epidermis by treating with Dispase II solution (Roche, Mannheim, Germany) overnight at 4 °C. The separated dermis was frozen and milled in liquid nitrogen. The powder was recovered in a 0.5 mL Tris (0.1 M) CaCl₂ (4 mM) buffer (pH 8.6) and centrifuged at 12000× *g* for 30 min at 4 °C. The supernatant was removed, and the pellet was treated with 1.2 mL of chloroform/methanol (2:1) and shaken gently for 30 min at room temperature. The defatted, dried skins were submitted to proteolytic digestion with 1 mL of protease from *Streptomyces griseus* (Sigma-Aldrich, Tokyo, Japan). This solution was incubated for 24 h at 50 °C and boiled for 10 min at 100 °C. The suspension was centrifuged at 12,000× *g* for 10 min at 4 °C. The supernatant was stored at −80 °C until analysis. Hyaluronic acid (HA) quantification was performed using enzyme-linked immunosorbent assay with a hyaluronic acid measurement kit (R&D Systems, Minneapolis, MN, USA).

### 4.10. Statistics

Statistical analysis data were presented as mean ± SEM. Statistical comparisons were carried out between control and various groups using Bonferroni’s test for multiple comparisons of one-way analysis of variance (ANOVA) using GraphPad Prism 5.0 (GraphPad Software Inc., San Diego, CA, USA). *P* values less than 0.05 were considered statistically significant.

## Figures and Tables

**Table 1:** Description of the experimental groups.

Group	UVB	Sample Dose
Normal	−	−
UVB	+	−
CP 500	+	500 mg/kg
CP 1000	+	1000 mg/kg
NAG	+	500 mg/kg

**Table 2:** Primer sequences used for quantification of gene expression.

Gene	Sequence
*HAS1*	forward: 5′-CTATGCTACCAAGTATACCTCG-3′
	reverse: 5′-TCTCGGAAGTAAGATTTGGAC-3′
*HAS2*	forward: 5′-GAGCACCAAGGTTCTGCTTC-3′
	reverse: 5′-CTCTCCATACGGCGAGAGTC-3′
*Hyal 1*	forward: 5′-TCATCGTGAACGTGACCAGT-3′
	reverse: 5′-GAGAGCCTCAGGATAACTTGGATG-3′
*Hyal 2*	forward: 5′-GCAGGACTAGGTCCCATCATC-3′
	reverse: 5′-TTCCATGCTACCACAAAGGGT-3′
*GAPDH*	forward: 5′-CCATGGAGAAGGCTGGGG-3′
	reverse: 5′-CAAAGTTGTCATGGATGACC-3′
